# Does the COVID-19 Pandemic Impact Reproductive Health?

**DOI:** 10.1055/s-0040-1718442

**Published:** 2020-11-30

**Authors:** Ana Laura Carneiro Gomes Ferreira, Maria Suely Medeiros Correa, Evelyne Nascimento Pedrosa, Flavia Anchielle Carvalho da Silva, Manuela Freire Hazin-Costa, Ariani Impieri Souza

**Affiliations:** 1 Instituto de Medicina Integral Professor Fernando Figueira, Recife, PE, Brazil; 2 Universidade de Pernambuco, Recife, PE, Brazil; 3 Faculdade Pernambucana de Saúde, Recife, PE, Brazil; 4 Universidade Federal de Pernambuco, Recife, PE, Brazil

Dear Editor,

Globally, governments have had to adapt and prioritize resources to the COVID-19 pandemic by providing health care for millions who have become ill of the virus. The stress that the COVID-19 pandemic imposed in the health systems forced authorities to order self or compulsory quarantine, even lockdown in some developing countries, restricting people's mobility to reduce the spread of the virus. Facing this new scenario, some reproductive health services have been closed down. 1 2 3

Moreover, the provision of basic contraception counselling, the delivery of contraceptive supplies and services have been interrupted. Women also fear about COVID-19 exposure and, due to the mobility restrictions, they cannot come to the family planning clinics and continue using their usual contraceptive method, which instead they may be using a less effective short-term method, or may have just interrupted the use of contraception altogether. 1 2 4 5

Evidence from previous large infectious outbreaks reinforce the adverse effects of the epidemic in sexual and reproductive services if the government does not ensure these essential services to remain open. Family planning services are available for all women and these services can improve the population's health, quality of life and strengthen both health services and the economy.

Six months of family planning interruption in low- and middle-income countries could result in 47 million women unable to use modern contraceptive methods, leading to an additional 7 million unintended pregnancies according to data released by the United Nations Sexual and Reproductive Health Agency (UNFPA). Unintended pregnancies can contribute to unfavorable outcomes ranging from unsafe abortion to severe pregnancy complications, which increases the maternal and newborn morbidity and mortality rates. 5 6 7

Considering sexual and reproductive services as essential health services, ensuring sustainable access to modern contraception and family planning services could be one strategic response to mitigate the impact of COVID-19 on women's health, as well as the impact on the wellbeing of families. 8 9 10

As family planning services are essential and the interruption of these services are in fact related to the pandemic, governments, health authorities and nongovernmental organizations must adopt a new way of providing contraceptive services, such as telehealth to support the continuity of contraceptive access. Technological strategies on care that require no personal contact, enhancing the use of telemedicine for counseling through cellphone apps and among others, are feasible alternatives. The use of social media for contraception, education and counseling, referral, and screening new patients, based on medical eligibility, has been advised. 4 8 9 10 11

New prescriptions and multimonth refills may be ensured for women who use oral contraceptives, contraceptive patches, or vaginal contraceptive rings, if there are no evident contraindications. Informing patients about emergency contraception includes advanced prescription options through electronic signature in which this may be another service to be provided. 4 8 9 10 11

Counselling new users to choose methods based on progesterone alone and non-hormonal methods is very prudent, especially at this time, when face-to-face contact is discouraged. Women desiring injectable contraception should be counselled to look for tutorial videos on Depot Medroxyprogesterone (DMPA) self-injection. 10 11 12 13

We need to continue encouraging new users to choose long acting highly effective, reversible contraceptives (LARCs), to ensure a safe procedure with scheduled visits, organizing the waiting room according to social distancing precautions and measures. We have to reassure LARC users that the removal can be delayed, due to its extended effectiveness beyond the labeled duration. 10 11 12 13 14

The International Federation of Gynecology and Obstetrics (FIGO), through its Contraception and Family Planning Committee, calls for the increased use of LARCs, emphasizing they do not require regular clinic visits and contacts with providers. 14

Another important cost-benefit strategy to face the interruption of contraception during the pandemic is counseling and access to LARCs postpartum or postabortion, ensuring the insertion of intrauterine dispositive/implants before hospital discharge or administrate DPMA if women desire to. 8 13 15

Commitment, resources and public support for Reproductive Health Services, in ensuring that women, adolescents and men can access safe and affordable contraceptive methods should be sustained during and beyond the COVID 19 pandemic. 8 9 16

The form these services are provided and to be adapted, new needs and circumstances should be considered by ensuring quality and equity. 9 16

In this context of innovation, there is a need of further research to evaluate if this new strategy on family planning can meet the standards for effective contraceptive practices. The scientific community must provide evidence in reassuring their commitment to provide the best quality in care.

## References

[1] Ahmed Z Sonfield A The COVID-19 outbreak: potential fallout for sexual and reproductive health and rights [Internet] New York Guttmacher Institute 2020[cited 2020 May 31]. Available from:https://www.guttmacher.org/article/2020/03/covid-19-outbreak-potential-fallout-sexual-and-reproductive-health-and-rights

[2] International Planned Parenthood Federation COVID-19 pandemic cuts access to sexual and reproductive healthcare for women around the world [Internet] 2020[cited 2020 May 31]. Available from:https://www.ippf.org/news/covid-19-pandemic-cuts-access-sexual-and-reproductive-healthcare-women-around-world

[3] Guterres A Put women and girls at the centre of efforts to recover from COVID-19 [Internet] 2020[cited 2020 Jun 01]. Available from:https://www.un.org/en/un-coronavirus-communications-team/put-women-and-girls-centre-efforts-recover-covid-19

[4] Nanda K Lebetkin E Steiner M J Yacobson I Dorflinger L J Contraception in the Era of COVID-19 Glob Health Sci Pract 2020 8 02 166 168 10.9745/GHSP-D-20-00119 32312738 PMC7326510

[5] Riley T Sully E Ahmed Z Biddlecom A Estimates of the potential impact of the COVID-19 pandemic on sexual and reproductive health in low- and middle-income countries Int Perspect Sex Reprod Health 2020 46 73 76 10.1363/46e9020 32343244

[6] United Nations Population Funds (UNFPA), Avenir Health, Johns Hopkins University, Victoria University Impact of the COVID-19 pandemic on family planning and ending gender-based violence, female genital mutilation and child marriage [Internet] 2020[cited 2020 May 25]. Available from:https://www.unfpa.org/resources/impact-covid-19-pandemic-family-planning-and-ending-gender-based-violence-female-genital

[7] United Nations Population Funds (UNFPA) New UNFPA projections predict calamitous impact on women's health as COVID-19 pandemic continues [Internet] 2020[cited 2020 May 25]. Available from:https://www.unfpa.org/press/new-unfpa-projections-predict-calamitous-impact-womens-health-covid-19-pandemic-continues

[8] FIGO Contraception, Family Planning Committee Townsend J W Ten Hoope-Bender P Sheffield J In the response to COVID-19, we can't forget health system commitments to contraception and family planning Int J Gynaecol Obstet 2020 ••• 10.1002/ijgo.13226[ahead of print] PMC9087681 32415990

[9] American College of Obstetricians and Gynecologists Implementing Telehealth in Practice: ACOG Committee Opinion Summary, Number 798 Obstet Gynecol 2020 135 02 493 494 10.1097/AOG.0000000000003672 31977794

[10] Sully E A Biddlecom A Darroch J E Adding it up: investing in sexual and reproductive health, 2019 New York Guttmacher Institute 2020

[11] World Health Organization Medical eligibility criteria for contraceptive use [Internet] 5th ed. Geneva WHO 2015[cited 2020 May 29]. Available from:https://www.who.int/reproductivehealth/publications/family_planning/MEC-5/en/

[12] Kennedy C E Yeh P T Gaffield M L Brady M Narasimhan M Self-administration of injectable contraception: a systematic review and meta-analysis BMJ Glob Health 2019 4 02 e001350 10.1136/bmjgh-2018-001350 PMC6528768 31179026

[13] Bahamondes L Fernandes A Monteiro I Bahamondes M V Long-acting reversible contraceptive (LARCs) methods Best Pract Res Clin Obstet Gynaecol 2020 66 28 40 10.1016/j.bpobgyn.2019.12.002 32014434

[14] FIGO Committee for Contraception and Family Planning COVID-19 contraception and family planning [Internet] 2020[cited 2020 May 19]. Available from:https://www.figo.org/covid-19-contraception-family-planning

[15] Liberty A Yee K Darney B G Lopez-Defede A Rodriguez M I Coverage of immediate postpartum long-acting reversible contraception has improved birth intervals for at-risk populations Am J Obstet Gynecol 2020 222(4S):8860 8.86E11 10.1016/j.ajog.2019.11.1282 PMC7147501 31846612

[16] World Health Organization Telemedicine: opportunities and developments in Member States: report on the second global survey one eHealth 2009 [Internet] Geneva WHO 2010[cited 2020 May 29]. Available from:https://www.who.int/goe/publications/goe_telemedicine_2010.pdf

